# Calcaneal BMD Obtained by Dual X-Ray and Laser Predicts Future Hip Fractures—A Prospective Study on 4 398 Swedish Women

**DOI:** 10.4061/2010/875647

**Published:** 2010-06-10

**Authors:** Torkel B. Brismar, Imre Janszky, L. I. M. Toft

**Affiliations:** ^1^Division of Radiology, Department for Clinical Science, Intervention and Technology, Karolinska Institute, Karolinska University Hospital, Huddinge, 141 86 Stockholm, Sweden; ^2^Division of Public Health Epidemiology, Department for Public Health Sciences, Karolinska Institute, 17177 Stockholm, Sweden; ^3^Merck Sharp & Dohme, 192 78 Sollentuna, Sweden; ^4^Pfizer AB, Stockholm, 191 90 Sollentuna, Sweden

## Abstract

The predictive value of dual X-ray and laser (DXL) calcaneal BMD (BMD_DXL_) on hip fractures was prospectively studied in 4,398 females aged 55 to 99 years. The average follow-up period was 3 years and 11 months with a total of 17,270 person years. Fractures were identified from the national patient register. After inclusion, 130 females sustained a hip fracture. The age adjusted hazard ratio for T-score <−2.5 versus >−2.5 was 2.64. Of all patients who sustained a hip fracture 78% had a T-score of −2.5 or below. The annual hip fracture rate was 0.26% at T-scores ≥−2, but 1.5% at T-scores ≤−2.5. The area under curve for the model including calcaneal BMD_DXL_, follow-up time, and age to prospectively predict hip fractures was 0.84. *Conclusions*. Calcaneal BMD_DXL_ obtained by DXL Calscan predicts hip fractures and may therefore be suitable for diagnosing osteoporosis and for predicting fracture risk.

## 1. Introduction

The state of the art technique to diagnose osteoporosis is dual energy X-ray absorptiometry (DXA) of femoral neck. Numerous studies have demonstrated the predictive value of femoral neck BMD and the risk of future hip fractures [1–3]. Bone densitometers that measure the BMD of the femoral neck do however require skilled personnel and they are often localized to university hospitals and dedicated osteoporosis centres. To make bone densitometer scans more available and less expensive peripheral scanners have been developed. These have also been shown to predict future fractures [4–8]. To our knowledge there has not been any comparative study showing significant superiority of femoral neck BMD over calcaneal BMD. In spite of the possible advantages of calcaneal BMD; they have still not been recommended for wider clinical use. One reason might be the didactic problem that calcaneus is not a bone that fractures due to osteoporosis. On the other hand, calcaneus consists mainly of trabecular bone and should therefore be affected by a negative bone balance at an earlier stage.

**Figure 1:**
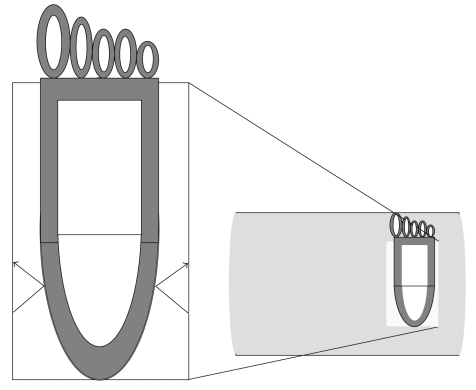
A schematic drawing of the DXL equipment and the placement of the foot. The black lines show how the laser beams are reflected at the surface of the skin, not at the calcaneal bone.

When performing standard DXA the relative proportion of bone and tissue is calculated by using two different X-ray energies (a two-component model). Because the absorption of radiation is lower in fat compared to the soft tissue, the fat content in the measured region is assumed to be constant at DXA. This is a limitation of standard DXA, because the calcaneal fat content varies between individuals [9] and probably also with age. The technique of calcaneal bone densitometry has been further improved by combining the two X-ray energies of DXA with a laser measurement of heel thickness (Figure 1) [9, 10]. This technique is most often called dual X-ray and laser (DXL). The measurement of heel thickness makes it possible to obtain a third parameter and the separation of bone mineral, lean tissue; and adipose tissue (a three-component model). The misclassification of bone into soft tissue (or vice versa) can thereby be reduced. However, the clinical advantage of this technique does require further evaluation. Because the BMD obtained using DXL is different from that obtained using standard DXA; the obtained BMD will be denoted BMD_DXL_ in this article. 

The aim of this study was to evaluate the predictive value on future hip fractures of calcaneal BMD_DXL_.

## 2. Material and Methods

This prospective clinical study was conducted during the period January 1, 2000 to December 31, 2007 at 19 healthcare units and outpatient clinics in Sweden (see Acknowledgments). The study was approved by the ethical committee and in part sponsored by the pharmaceutical company Merck Sharp & Dohme (rental of scanners and education of participating centers). Patients were informed by the staff and by posters and information sheets in the waiting room. Inclusion criteria were patients aged 55 to 99 years with a clinical suspicion of osteoporosis. Patients with a hip fracture after January 1, 1990 but before inclusion in the study were excluded. Date of death provided by the national cause of death register was used as censoring time. 

Calcaneal BMD_DXL_ was measured by dual X-ray and laser (DXL) to obtain the bone mineral density in g/cm^2^ (DXL Calscan or Calscan DEXA-T, Demetech AB, Stockholm, Sweden). Some of the centers continuously measured both feet, while other centers for efficacy reasons measured one foot. When both feet were measured (approx 1/4 of the cases) the average BMD_DXL_ of the two feet was used. In a previous publication [11], on a part of the patient material in this study, no statistically significant difference between the right and left foot could be observed when both feet were measured in 334 individuals. The centralized health care system in Sweden provides virtually complete follow-up information for all patients by matching their unique 10-digit person identification numbers to the patient and other health registers. By matching the database of all included patients with the national patient register, all hip fractures could be identified. 

### 2.1. Statistics

T-score calculations were based on a published reference population [12]. The average decrease in BMD_DXL_ with age was calculated using linear regression. The standard deviation from that average decrease was assumed to be constant with age and it was used as the basis for the calculations of Z-score (i.e., the number of standard deviations the individual BMD_DXL_ is from the average age adjusted BMD_DXL_). For comparisons between groups, Student's *t*-test was used. To calculate annual hip fracture rate all individuals were ordered with increasing T-score. For every individual; the observation time, corrected for death, was then noted. By using a sliding sum, the number of fractures that occurred for the surrounding 1000 observation years were counted. By dividing that number with 1000 and expressing it in percentage annual hip fracture rate could be estimated at that *t*-value. At T-scores <−4.5 the number of patients was too low to allow a total number of 1000 observation years and the 1000-observation-year base was then continuously adjusted. 

Age adjusted Cox proportional hazard models were used to examine the association between T-score and hip fracture. As in other studies, T-score was included as a continuous variable and the hazard ratio (HR) was calculated for each unit decrease. Alternatively, T-score was also categorized as ≤−5, >−5 and ≤−4, >−4 and ≤−3, >−3 and ≤−2, >−2 and ≤−1, and >−1. The latter approach allowed us to model the association between T-score and hip fracture without taking an assumption about the nature of the relationship, that is, linearity. The proportionality of hazards was evaluated using formal two-sided tests of interaction with time and logarithm of time. There was no evidence against the proportionality of hazards.

## 3. Results

**Figure 2:**
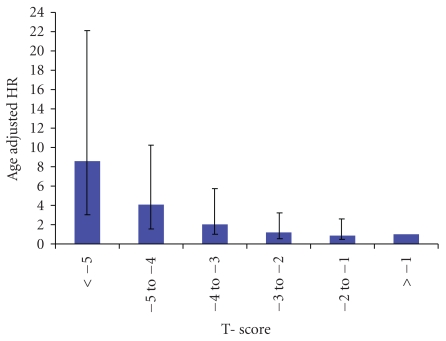
The age-adjusted hazard ratio with different T-score categories compared against the hip fracture rate at T-score >−1.

In total 4,398 females with an average age of 70 years (SD 9) were included. The average follow-up period was 3 years and 11 months with a total of 17,270 person years. In 1,140 females both feet were measured (26%) and in the remaining 3,528 females BMD_DXL_ was obtained from either the right (*n* = 1,221) or the left foot (*n* = 2,037). The standard deviation from the age adjusted BMD_DXL_ that is, the Z-score, was 0.073 g/cm^2^. The number of patients per decade, observation period, BMD_DXL_, T-scores and number of fractures are shown in Table 1. The corresponding stratified data in terms of T-score have been shown in Table 2. In total, 130 females sustained a hip fracture after inclusion in the study. Individuals suffering from hip fracture were significantly older than those who did not suffer from a hip fracture during the study; mean age at recruitment was 78.1 (SD 8.1) years compared to 69.3 (SD 8.7 years). Their average T-score was significantly lower than that of nonfractured individuals −3.50 (SD 1.4) compared to −2.16 (SD 1.3). The difference was significant also after adjustment for age (i.e. calculation of the Z-score), −0.62 (SD 1.1) versus 0.02 (SD 0.99). Of the 130 patients who sustained a hip fracture 101 (78%) had a T-score of −2.5 or below. No patient with a T-score >0.13 sustained a hip fracture. The area under curve (AUC) of calcaneus to predict hip fractures was 0.84 (0.81 to 0.88, 95% confidence interval) when adjusted for age and follow-up time. The age adjusted hazard ratio for an individual with a T-score <−2.5 versus an individual with a T-score >−2.5 was 2.64 (95% CI: 1.7–4.1, *P* < .0001) The hazard ratio for each unit decrease in T-score was 1.74 (1.49–2.04). The hazard ratio for the categorized T-scores is shown in Table 3 and in Figure 2.

## 4. Discussion

According to the WHO, osteoporosis is defined as a T-score in hip, spine or radius of −2.5 or less. When making peripheral measurements, for instance in calcaneus, it may be discussed at which limit the threshold for diagnosing osteoporosis should be set [12, 13]. If the threshold of −2.5 is applied to our data, DXL calcaneal T-score successfully predicts 78% of the patients who will suffer from a hip fracture during the observation period. This is significantly greater than previously published data using femoral neck BMD or lumbar spine BMD, where less than 50% of the hip fracture patients were identified [14]. However, in that study the females were ≥65 years and randomly selected from an American population, while our population were Swedish females ≥55 years with suspected osteoporosis. The proportion of patients with a T-score <−2.5 was therefore greater in our study (40% compared to 17%). 

The hazard ratio for each unit decrease in T-score (HR) was 1.74. This ratio is lower than the HR of 2.6 published for BMD of the hip [15]. Our material was however skewed so that the included population had a suspicion of osteoporosis. The Swedish population does also have a higher risk of hip fracture compared to non-Scandinavian populations. This higher underlying risk may itself explain the observed lower relative risk in our study [16]. Our analyses with T-score categories suggested that the relationship was not linear (Figure 2). The increased hazard for hip fractures in our study was confined to those with a T score ≤−3. It is therefore difficult to compare the HR obtained in this study with that of other studies. To the best of our knowledge, linearity has not been systematically examined in previous studies on HR and hip BMD. 

**Figure 3:**
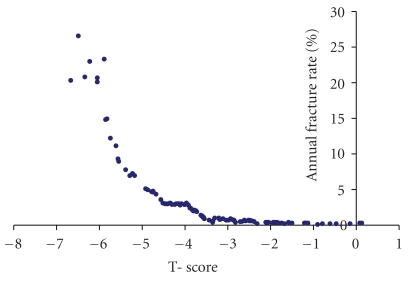
The annual hip fracture rate at different T-scores. Every dot represents one fracture. The annual fracture rate is estimated with greater uncertainty for T-scores below −4.5 due to a limited number of individuals.

A general recommendation to calculate the 10-year risk has been proposed [17]. In our study the observation period was almost 4 years. We have therefore chosen to calculate the annual fracture rate and we intend to repeat the observations when we have a 10-year observation period. The annual fracture rate could theoretically be used to make 10-year risk estimations, but it would most probably underestimate the 10-year risk because bone mineral density decreases with increasing age and the risk for fracture increases with age. The annual rate of sustaining a hip fracture was 0.26 % at T-scores of −2 or greater, which can be compared to 1.5% in individuals with a T-score of −2.5 or less (a 5.6-fold increase in relative rate). When calculating the age adjusted hazard ratio for those below the T-score <−2.5 threshold versus those above the threshold was 2.64. At calcaneal T-scores below −3.5 the fracture risk increased dramatically (Figure 3) and quickly reached levels where also highly intensive counteractive measures are strongly indicated. 

In a study by Kanis et al. a 10-year risk for hip fracture of 7.5% has been proposed as a justification threshold for treatment [18]. When taking also other osteoporotic fractures into account it was proposed that threshold could be decreased to 5%. In our material, based on annual fracture rate, the 5% threshold should be reached approximately at a T-score of −2.5. To our knowledge; there has not been any fracture preventive study published based on populations selected on the basis of low calcaneal BMD or calcaneal BMD_DXL_. It is therefore not yet possible to recommend calcaneal BMD or calcaneal BMD_DXL_ for monitoring treatment effect. However, in a comparative study on oral and intravenous bisphosphonates, the gain in BMD was greater in calcaneus than in femoral neck and lumbar spine [19]. This suggests that calcaneal BMD might be more sensitive to changes in bone mineral metabolism.

**Figure 4:**
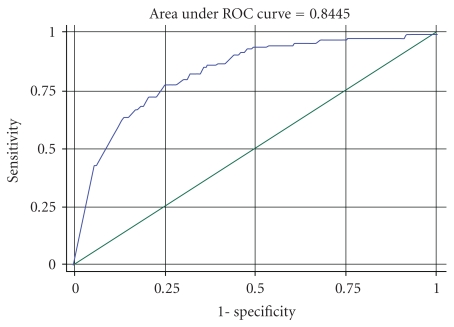
The sensitivity of DXL Calscan to predict hip fractures as a function of decreasing specificity.

In 1993; Cummings et al. published a comparative study on single X-ray absorptiometry (SXA) of calcaneus and DXA of femoral neck for prediction of hip fractures [4]. In the present study, the area under curve (AUC) of calcaneus to predict hip fractures (0.84, Figure 4) was higher than either AUC of femoral neck (0.76) or that of calcaneus (0.70) in Cummings' study. The better outcome in our study compared to that of Cumming's is probably due to the use of a three-component model when quantifying BMD. Errors in BMD estimation from interindividual variation in amount of calcaneal fat tissue can thereby be avoided [9]. The obtained AUC to predict hip fractures obtained in our study was also greater than that published by Hans et al. who in the EPISEM prospective cohort of 12,958 Elderly Women observed an AUC of about 0.68 [20], but the average age in that study was greater and the age distribution was smaller (78 years (SD 4) compared to 70 years (SD 9)), which affects AUC unfavourably.

No cross-calibration and no external reference phantom was used in this study. This might have obscured our data and may theoretically reduce the calculated HR. However, in a recently published reproducibility study of the equipment used in our study the interequipment short-term reproducibility expressed as average difference from mean BMD_DXL_ was less than 0.008 g/cm^2^, equivalent to 0.13 in T-score [11]. The negative influence of not using cross-calibration and an external reference phantom in our study was therefore considered minor. 

The hip fractures in this study were identified by cross-linking with the national patient register. This is a major strength of our study, because the coverage of this registry is about 99.7%.

## 5. Conclusion

The age adjusted AUC of DXL of calcaneus to predict future hip fractures was 0.84, which is better than that previously reported for DXA of the femoral neck. Of the patients who sustained a hip fracture 78% had a DXL T-score of <−2.5. DXL of calcaneus may therefore be suitable for diagnosing osteoporosis and for predicting fracture risk.

## Figures and Tables

**Table 1:** The number of individuals per decade, observation years, BMD_DXL_, T-score with 1 standard deviation within parenthesis, number of fractures and the annual hip fracture rate regardless of BMD_DXL_.

Age	Number	Observation years	BMD_DXL_(g/cm^2^)	T-score	Fractures	Annual fracture rate (%)
55–59	772	3208	0.391 (0.074)	−1.5 (1.2)	5	0.16
60–64	816	3292	0.378 (0.071)	−1.7 (1.1)	6	0.18
65–69	717	2956	0.358 (0.070)	−2.0 (1.1)	9	0.30
70–74	755	2986	0.336 (0.076)	−2.4 (1.2)	20	0.67
75–79	724	2776	0.318(0.075)	−2.7 (1.2)	27	0.97
80–84	437	1488	0.292(0.073)	−3.1 (1.2)	38	2.55
85–99	177	563	0.259 (0.084)	−3.6 (1.4)	25	4.44
All	4398	17270	0.347(0.082)	−2.2 (1.3)	130	0.75

**Table 2:** The number of individuals with different T-scores, their mean age with 1 standard deviation within parenthesis, the number of fractures, observation years and the annual fracture rate.

T-score	Number	Mean Age	Fractures	Observation years		Annual fracture rate
<−6	12	82 (8)	6	24		25%
−6<−5	57	81 (9)	11	175		6.3%
−5<−4	315	78 (7)	34	1134		3.0%
−4<−3	772	73 (8)	34	3128		1.1%
−3<−2	1297	70 (8)	25	5174		0.48%
−2<−1	1254	67 (8)	13	4855		0.27%
−1<0	494	65 (7)	5	1928		
0 < 1	153	64 (7)	2	594		
1 < 2	22	64 (8)	0	112		
2 < 3	11	63 (6)	0	65	2779	0.25%
3 < 4	4	63 (6)	0	30		
4 < 5	6	66 (1)	0	42		
5 < 6	1	56	0	8		
<−2.5	1769	74 (8)	101	6919		1.5%

**Table 3:** The age-adjusted hazard ratio of hip fracture with different T-score categories compared against T-score >−1.

T-score	Hazard Ratio	95% confidence interval	
<−5	8.6	3.3	22.3
−5 to −4	4.1	1.7	9.6
−4 to −3	2.0	0.88	4.7
−3 to −2	1.2	0.51	2.8
−2 to −1	0.86	0.34	2.2
